# Useful Information

**Published:** 1879-10

**Authors:** 


USEFUL INFORMATION.—One of our readers, writing from a prominent city in the northern part of the State, objects to allowing so much space in the JOURNAL AND EXAMINER, to the proceedings of medical societies; and alluding particularly to the record of proceedings of the Illinois State Medical Society, contained in the July number, asks what possible use it can be except to enable those who were present "to sec their names in print." Thinking that this writer may be representative of a class, we will answer his question with great pleasure. The record of proceedings as published are of use; *first*, as the means of informing a large number of members of the profession that such an organization as the Illinois State Medical Society exists; that it holds its meetings annually, the last of which was in the city of Lincoln; that its last meeting was attended by delegates and members from a large number of county and other societies in the State, and that many reports and papers of value were read, discussed and referred for publication in a volume: *second*, it informed a large circle of readers what action was taken on several important questions, such as the proper mode of determining questions of insanity and care for the insane; the sanitary improvement of public schools; the maintainance of a proper standard of education for the profession, etc: *third*, by stating what reports and papers were read, and by whom, it enabled each one of our readers to see whether there were any that they would desire to have; and by giving the names of the officers elected for the ensuing year, each reader would know to whom application could be made for full copies of the transactions when published; and, *fourth*, by giving the names of the new standing committees, and the time and place for holding the next annual meeting, it enables each reader of the JOURNAL AND EXAMINER to know to whom they should send any valuable facts coming under their observation during the year, that they may contribute to the fund of professional knowledge, and assist in making the next meeting more interesting and profitable, and thereby advance the general interests of the profession. We are sorry that our friend, or any other member of the profession in this State, should not be able to appreciate any of these important *uses* to be made of the simple record of proceedings of the State Medical Society. We hope he will profit by hints here given and, not only be induced to send for the full volume of Transactions which is now just through the press and ready for distribution, but will also find his way to the next annual meeting as a delegate from the city or county society where he lives. We are certain he would be greatly profited by doing so, besides having the pleasure of seeing his own name in print.

